# The complete mitochondrial genome of the Korean endemic species *Cobitis hankugensis* (Kim, Park, Son & Nalbant, 2003)

**DOI:** 10.1080/23802359.2021.2005496

**Published:** 2021-12-10

**Authors:** Soo Rin Lee, Eun-Bi Kim, Yunji Go, Yuan Kang, Md. Jobaidul Alam, Kyung Su Kim, Sapto Andriyono, Hyun-Woo Kim

**Affiliations:** a Industry 4.0 Convergence Bionics Engineering, Pukyong National University, Busan, Republic of Korea; b Department of Marine Biology, Pukyong National University, Busan, Republic of Korea; c Department of Fisheries, Ministry of Fisheries and Livestock, Dhaka Bangladesh; d Gyeongsangnam-do Freshwater Fish Research Center, Miryang, Republic of Korea; e Marine Integrated Biomedical Technology center, The National Key Research Institutes in Universities, Busan, Republic of Korea; f Fisheries and Marine Faculty, C Campus Jl, Universitas Airlangga, Surabaya, Indonesia

**Keywords:** Next generation sequencing, *Cobitis hankugensis*, mitochondrial genome, Cobitidae

## Abstract

As one of efforts to conserve a genetic resource of the endemic cobitid species in the Korean peninsula, the complete mitogenome of *Cobitis hankugensis* (Kim, Park, Son & Nalbant, 2003) was determined using Illumina MiSeq system. The circular mitogenome was 16,557 bp length and encoded 13 protein-coding genes (PCGs), two ribosomal RNA genes, 22 tRNA genes, and a control region. Only the COX1 gene was identified with an aberrant initiation codon GTG, and an incomplete termination codon (T—/TA–) was identified in six PCGs including COX2, COX3, ND2, ND3, ND4, and Cytb genes. Phylogenetic analysis using 30 mitochondrial genomes belonging to Cobitidae, Botiidae, and Gyrinocheilidae showed that the highest identity (92.38%) with *Kichulchoia brevifasciata* (NC_027166). The complete mitogenome of *C. hankugensis*, an endemic species in Korea, will provide fundamental data on the evolutionary relationship of Cobitidae species.

Fish in the family Cobitidae are widely distributed in the Palearctic region (Šlechtová et al. 2008). Among 146 species currently reported in the family, 17 species (5 genera) are known in Korean rivers (Kim 2009). As one of endemic species in Korean peninsula, *Cobitis hankugensis* (Kim, Park, Son & Nalbant, 2003) mainly inhabits in Nakdong River and Hyongsan River as well as their tributaries. Due to the morphological similarity, *C. hankugensis* often confused with its relatives, including *C. tenia* and *C. sinensis* (Kim 2009). In particular, interspecific hybridization between *C. hankugensi*s and its relative, *Iksookimia longicorpa* has been detected (Perdices et al. 2016; Kwan et al. 2019). Besides, many foreign species in the genus have been introduced for their aquaculture. Therefore, it is urgent to secure the native genetic resources of cobitid fish in Korea for its sustainability.

*Cobitis hankugensis* were collected from a tributary of Nakdong River, South Korea (N35°32’20.96”, E128°6’27.73”) in 2018. The mitochondrial DNA was isolated using the commercial mitochondrial isolation kit (Abcam, USA). Species identification was conducted by the morphological characteristics (Kim et al. 2003; Kim 2009) and its nucleotide sequence identity of COI region (99.02%) with the reference sequence of *C. hankugensis* (EU670772) in the database. The specimen and its DNA are stored at the Marine Biodiversity Institute of Korea (https://www.mabik.re.kr/html/en/, Ha Yeun Song, and hysong@mabik.re.kr) under the number MABIK GR00004770. For the high-throughput sequencing, the mitochondrial DNA was sheared by Covaris M220 Focused-Ultrasonicator (Covaris Inc., USA), which was further used for constructing a library using the TruSeq^®^ RNA library preparation kit V2 (Illumina, USA). The constructed library was sequenced by the Illumina MiSeq sequencing platform. After removal of the low-quality reads and adapter region, the trimmed reads were assembled and annotated by using Geneious Prime 2021.0.3 (https://www.geneious.com) under the default parameter settings. The secondary structures of 22 tRNAs were predicted by the tRNAScan-SE online software (Lowe and Chan 2016).

The complete circular mitogenome of *C. hankugensis* (MZ339224) was 16,657 bp in length, which encoded the 13 protein-coding genes (PCGs), two ribosomal RNA genes, 22 tRNA genes, and a control region. The overall AT and GC contents were 57.84% and 42.16%, respectively, which is similar to other freshwater fish taxa. Except for ND6 gene and eight tRNAs (Gln, Ala, Asn, Cys, Tyr, Ser, Glu, Pro), a total of 12 PCGs, two ribosomal RNAs, and 14 tRNAs were located on the heavy strand (H-strand). The control region (929 bp) was identified between tRNA-*Pro* and tRNA-*Phe*. Twelve PCGs were started with typical initiation codon (ATG), except for COX1 (GTG). Transcription of six PCGs were predicted to be terminated with the typical stop codons (TAA), while the incomplete termination codons (T–/TA—) were identified in six PCGs including COX2, COX3, ND2, ND3, ND4, and Cytb genes.

In order to confirm the evolutionary relationship of *C. hankugensis* with its relatives, total of 31 complete mitochondrial genomes were obtained from the GenBank database (https://www.ncbi.nlm.nih.gov/genbank/) and a phylogenetic tree was constructed (Figure 1). Two sister species, Gyrinocheilidae *Gyminocheilus pennnocki* (NC_031544) and Botiidae *Leptobotia microphthalma* (NC_024049) were selected as outgroup members. The nucleotide sequences of 13 PCGs were aligned by using MAFFT v7.48 with the L-INS-I algorithm (Katoh et al. 2019). The Maximum likelihood (ML) phylogenetic analysis was performed with 1,000 bootstrap replicates based on the GTR + F+R4 model in the IQ-TREE2 package version 2.1.3 (Minh et al. 2020). As a result, *C. hankugensis* showed the highest nucleotide sequence identity (92.38%) with *Kichulchoia brevifasciata* (NC_027166) followed by the *Cobitis biwae* (91.08%, NC_0 27663) (Figure 1). This result strongly supported the previous biogeographical analysis of cobitid species, in which those species have been evolved in South subdistrict (Kwan et al. 2018).

**Figure 1.**
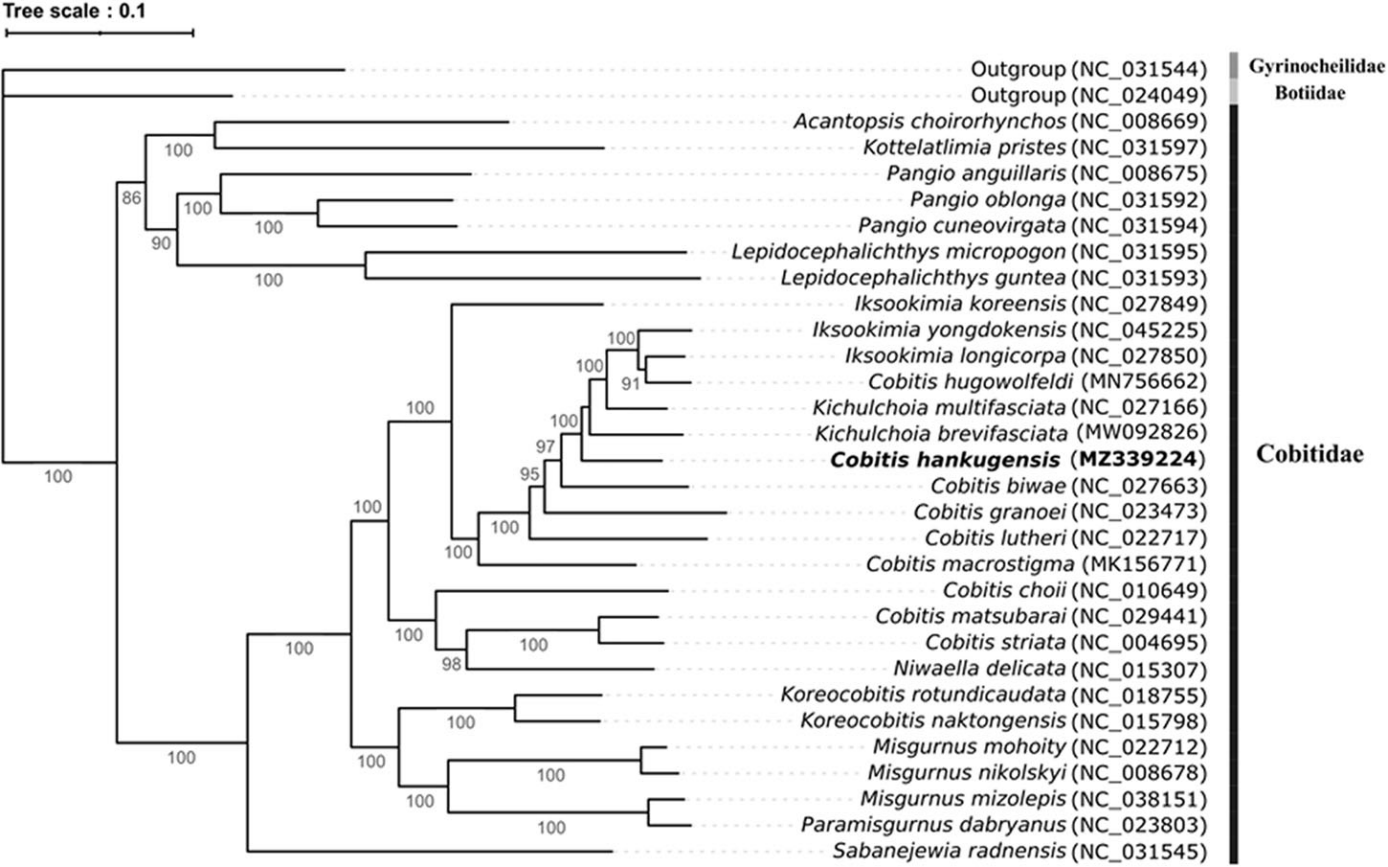
Phylogenetic relationship of *Cobitis hankugensis* in the family Cobitidae.

A phylogenetic tree was based on the complete mitogenomes in the family Cobitidae using the IQ-TREE2 package version 2.1.3 by Maximum likelihood (ML) algorithm. Number at each node indicates bootstrap replications. The GenBank accession numbers were shown followed by each species scientific name.

## Data Availability

The mitogenome data that support the findings of this study are available in GenBank of NCBI at: https://www.ncbi.nlm.nih.gov/nuccore/MZ339224. Associated accession numbers BioProject: PRJNA732999, BioSample: SAMN19357315, and SRA: SRR14663551 are available.
